# Multilayer Patterning of High Resolution Intrinsically Stretchable Electronics

**DOI:** 10.1038/srep25641

**Published:** 2016-05-09

**Authors:** Klas Tybrandt, Flurin Stauffer, Janos Vörös

**Affiliations:** 1 Institute for Biomedical Engineering ETH Zurich, ETZ F76 Gloriastrasse 35 8092 Zurich Switzerland

## Abstract

Stretchable electronics can bridge the gap between hard planar electronic circuits and the curved, soft and elastic objects of nature. This has led to applications like conformal displays, electronic skin and soft neuroprosthetics. A remaining challenge, however, is to match the dimensions of the interfaced systems, as all require feature sizes well below 100 μm. Intrinsically stretchable nanocomposites are attractive in this context as the mechanical deformations occur on the nanoscale, although methods for patterning high performance materials have been lacking. Here we address these issues by reporting on a multilayer additive patterning approach for high resolution fabrication of stretchable electronic devices. The method yields highly conductive 30 μm tracks with similar performance to their macroscopic counterparts. Further, we demonstrate a three layer micropatterned stretchable electroluminescent display with pixel sizes down to 70 μm. These presented findings pave the way towards future developments of high definition displays, electronic skins and dense multielectrode arrays.

Adaptation of devices to curvilinear surfaces and to soft and deformable systems requires stretchability^1^,^2^. Recent progress has resulted in applications like conformal displays^3^,^4^, electronic skin^5^,^6^, soft neuroprosthetics^7^,^8^,^9^ and cardiostimulating implants^10^. Also, new stretchable components such as transistors^11^,^12^ and batteries^13^ are under development. A remaining challenge, however, is to match the dimensions of the interfaced systems, as for example both high definition displays and neural recording electrodes require feature sizes well below 100 μ m. Intrinsically stretchable nanocomposites^1^,^14^,^15^,^16^,^17^,^18^,^19^ are attractive in this context, as the mechanical deformations occur on the nanoscale and thus potentially allows for far reaching miniaturization, which is not the case for macroscopic geometrical constructs like serpentines. The composites typically comprise an elastomer loaded with a conductive filler, e.g. carbon nanotubes^18^, graphene^20^, (nano)particles^14^,^21^,^22^ or metal nanowires^16^,^17^. Miniaturization of stretchable conductors may seem like a trivial task, but strain induced defects and inhomogeneities which may be tolerable for macroscopic samples^16^ are fatal for narrower features^23^. Only a few reports have been published on the patterning and characterization of stretchable conductors with line widths < =  500 μ m (Fig. 1a)^9^,^22^,^23^,^24^,^25^,^26^, and none of these combines small dimensions with high performance. The challenge is thus to develop a method which can produce arbitrary patterns of high resolution, low sheet resistance and high stretchability (lower left corner of Fig. 1a). Preferably the method should also be compatible with multilayer fabrication of elastomer substrates, which are sensitive to organic solvents and many etching solutions^27^,^28^. In order to meet these criteria, herein we report on a filtration based method for the fabrication of high resolution multilayer stretchable devices. Filtration is a well-established method to create films of carbon nanotubes and silver nanowires (AgNWs), although up till now only low resolution patterns have been reported^16^,^24^. Here we improve the resolution of the method and achieve outstanding electromechanical performance for our micropatterned conductors (Fig. 1a). Interestingly, there are so few defects in the conductors that 20 mm long and 30 μ m wide tracks electromechanically behave similarly to macroscopic tracks. Further, we utilize this versatile method to fabricate a three layer stretchable matrix of electroluminescent pixels with outstanding fill factor and pixel size down to 70 μ m.

## Results and Discussion

**Figure 1:**
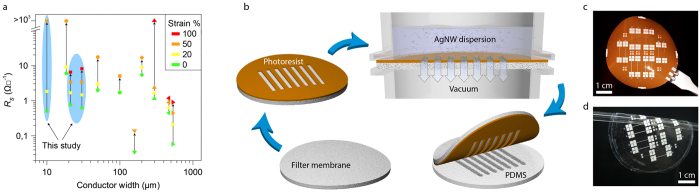
Stretchable conductor patterning. (**a**) Overview of published results on patterned stretchable composites with line widths < =  500 μ m (• Martinez *et al.*^25^, ▾ Larmagnac *et al.*^9^, ♦ Lacour *et al.*^26^, ▴ Moon *et al.*^23^, ◂ Matsuhisa *et al.*^22^, ▸Tybrandt *et al.*^24^). The sheet resistance (when available) for 0%, 20%, 50% and 100% strain for the different studies is plotted as a function of conductor width. (**b**) The membrane is patterned with a photoresist layer and the nanowires are deposited in the resist openings through vacuum filtration. The membrane is sequentially dried and put in contact with a semi-cured PDMS film under pressure and heat. Finally, the membrane is peeled off leaving the nanowire pattern on the PDMS surface. (**c**) The patterned membrane has a shiny non-sticky surface. (**d**) PDMS substrate with transferred nanowires. Features down to 20 μ m are transferred over the whole area.

The process starts by patterning a ma-N 490 photoresist layer on top of a hydrophilic PVDF membrane filter (Fig. 1b). The viscous resist is only partially absorbed into the membrane and the UV exposure mainly renders the top part of the resist insoluble, which allows for the development of high resolution patterns without clogged membrane pores. The resulting membrane has a shiny surface (Fig. 1c), which indicates that the resist forms an even layer on top of the pores. The thickness of the resist layer varies slightly over the membrane surface but is typically in the 3–5 μ m range (Supplementary Fig. 1). Two other photoresists, ma-P 1275 HV and SU-8 2100, were evaluated for the same purpose but without satisfying results. The positive ma-P 1275 HV resist left residues in the membrane pores even after extended development, which also resulted in non-vertical sidewalls (Supplementary Fig. 2). The SU-8 2100 resist was fully absorbed into the membrane, which made it impossible to achieve small open features due to the thickness and light scattering.

**Figure 2:**
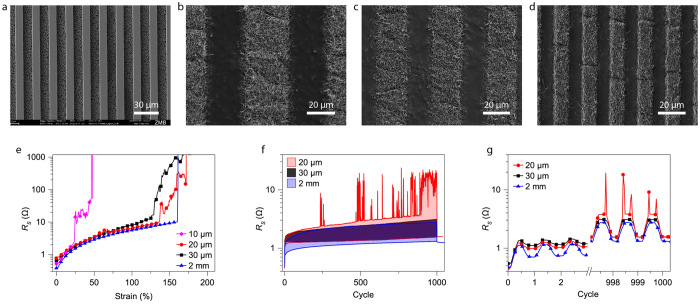
Characterization of AgNW patterns. (**a**) The 10 μ m photoresist lines have sharp edges and the unexposed photoresist have successfully been removed, leaving the pores in the membrane open. (**b–d**) AgNW tracks on PDMS of 30 μ m, 20 μ m and 10 μ m width, respectively. (**e**) Tracks of 20 mm length with different widths were stretched while measuring their resistance. All tracks had initially sheet resistance below 1 Ω /□ . The 20 μ m and 30 μ m tracks behaved similarly to the macroscopic 2 mm track when stretched. (**f**) Samples were cycled to 20% strain 1000 times. The lower resistance curve corresponds to no strain while the higher limit corresponds to 20% strain. After a few hundred cycles resistance spikes started to appear for the 20 μ m sample when stretched. (**g**) The resistance spikes for the 20 μ m sample appears in the stretched state. When the strain is released the resistance returns to the baseline value.

In the next step, the ma-N 490 patterned membrane is placed in the filtration setup and a dilute aqueous AgNW dispersion (15 ml) is sucked through the openings of the photoresist pattern by vacuum. Since the NWs follow the flow of the liquid they are deposited in the openings of the resist, with only few NWs deposited on top of the resist. The membrane is dried and hard baked as this prevents residual NWs, which are stuck on top of the resist, to be transferred. Finally, the membrane is pressed in contact with a semi-cured PDMS substrate, heated, and peeled off to complete the transfer. Figure 1d shows a two inch PDMS sheet with a transferred AgNW pattern. Feature sizes down to 20 μ m are typically transferred without any problems; smaller features are less reliably transferred but often work as well. The resolution limit of the method was evaluated by processing a set of test structures. Resist features down to 10 μ m with well-defined edges could readily be achieved (Fig. 2a) and locally it was even possible to achieve 5 μ m resolution (Supplementary Fig. 3). Next, we evaluated transferred AgNW patterns of 30 μ m, 20 μ m and 10 μ m line width (Fig. 2b–d). No defects were visible when a larger area was inspected (Supplementary Fig. 4a). A closer look at the 30 μ m tracks shows that the tracks are nicely separated with little nanowire residues in between (Fig. 2b). Interestingly, it seems like the nanowire tracks are partially penetrated with PDMS, which might explain the low amount of defects in the transfer, as this ensures good adhesion between the tracks and the substrate (Supplementary Fig. 4b,c). The 20 μ m tracks are intact but nanowire residues start to become a problem. Reducing the track width further down to 10 μ m exacerbate the problem. To determine whether the tracks were electrically isolated from each other, electrical measurements were performed on the test structures comprising four parallel 10 mm long lines with contact pads (Supplementary Fig. 5). Typically, the 30 μ m tracks were properly isolated (6 measured structures), the 20 μ m tracks had some shorts that could be melted away by applying 30 V, and the 10 μ m tracks were severely shorted (Supplementary Fig. 5). An important factor which limits the resolution is the conductor separation to AgNW length ratio. Here we need a ratio of three to achieve electrical isolation of tracks, which can be understood from the fact that this requires three residual AgNWs to line up to create a short, something which is quite unlikely as the density of transferred residual AgNWs are low. Also, it is very important not to have AgNW aggregates in the dispersion, as a few aggregates can ruin a finer pattern.

Electromechanical characterization was performed on 20 mm long test structures with varying track widths (Fig. 2e,f). The tracks were insulated with an additional PDMS layer, leaving only the nanowires at the contact pads exposed at the surface. The initial sheet resistance of the 20 μ m and 30 μ m tracks were 0.71 ±  0.24 Ω /□ and 0.74 ±  0.20 Ω /□ , respectively. This corresponds to a conductivity of about 5000 S/cm for the ~3 μ m thick AgNW layer (Supplementary Fig. 6). The resistance as a function of strain for the different samples is shown in Fig. 2e. When stretched, the resistance increases smoothly up to a certain point, after which a rapid increase with a lot of noise follows. This point occurs already at ~25% strain for the 10 μ m sample and at ~130% strain for the 20 μ m and 30 μ m samples. The 2 mm wide reference sample does not reach this point before it mechanically breaks at ~150% strain. This indicates that the transition is caused by local defects in the narrow tracks, while a few local defects don’t affect the macroscopic conductor. Even at 100% strain, the resistance of the 20 μ m and 30 μ m samples remains below 10 Ω /□ . The samples performance under repeated strain cycles was evaluated by applying 1000 cycles of 20% strain while measuring the resistance (Fig. 2f). The lower lines correspond to 0% strain while the upper lines correspond to 20% strain. The 30 μ m and 2 mm samples behave similarly and show stable performance. The resistance of the 20 μ m track starts to spike occasionally after a while when stretched, probably due to one or a few defects along the 20 mm long track. The spikes occur only locally during the strain cycle, after which the resistance returns to the normal range (Fig. 2g). Impressively, all samples remain below 2 Ω /□ after 1000 cycles. As narrow tracks are much more sensitive to local defects than wide tracks, the comparable performance indicates that the track quality is outstanding over the 20 mm long samples.

**Figure 3:**
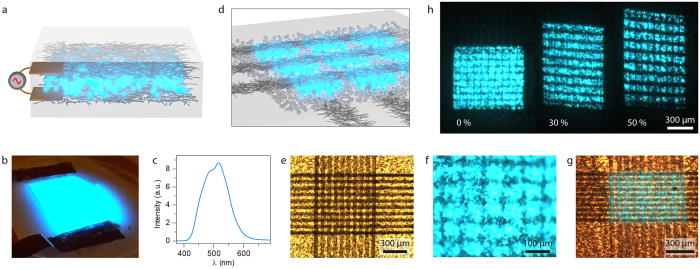
Electroluminescent matrix display. (**a**) Schematics of EL phosphor particles that are dispersed in PDMS between two AgNW electrodes. (**b**) The emission is uniform on the macroscopic scale with a measured luminance of 21 cd/m^2^. (**c**) The emission peak is centered at ~500 nm which gives a bluish light. (**d**) A matrix display configuration is achieved by sandwiching the EL particles between to grids of semi-transparent AgNW tracks. The electric field is strongest in the crossings of the tracks which defines the pixels. (**e**) A bright field image reveals that the EL particles are evenly dispersed over the surface. The two layers of AgNW tracks are also visible. (**f**) When the EL matrix is turned on the individual pixels of ~70 μ m in size are clearly visible. (**g**) When stretched the AgNW tracks widens in one direction and become narrower in the other direction. The EL emission from the crossing is visible. (**h**) Images of an EL matrix in the dark when stretched. The intensity goes down slightly with increasing strain.

The development of high resolution stretchable displays requires that several layers of stretchable conductors can be patterned in a multilayer configuration. Furthermore, at least one of the electrode layers needs to be transparent and the tracks have to be dense in order to obtain high resolution and fill factor. Electroluminescent (EL) phosphor is attractive for stretchable applications and recently the first stretchable panels were demonstrated^29^,^30^,^31^. The EL particles are dispersed in a dielectric layer and emit light when a high AC field is applied (Fig. 3a). As no direct contact is needed between the particles and the electrodes, no contact issues arise between the layers when stretched. Also, no oxygen or water barriers are needed for long term operation of EL phosphor displays which is very advantageous as the incorporation of barriers is an unresolved problem for stretchable electronics. First we constructed an unpatterned EL panel for characterization purposes (Fig. 3a,b). EL particles with diameter <7 μ m were dispersed in PDMS to a 1:1 weight ratio and spin coated on top of an AgNW layer. The top AgNW electrode was transferred and sealed by an additional layer of PDMS. The luminance of the display was 21 cd/m^2^ when operated with a standard battery driven inverter (168 V, 770 Hz). The color was bluish with intensity maxima around 500 nm (Fig. 3c). In the next step a matrix of EL pixels was fabricated by incorporating an EL-PDMS layer in between a grid of partially transparent AgNW electrodes (Fig. 3d). The bright field image in Fig. 3e shows that the EL particles were evenly spread out throughout the 20 μ m thick layer (Supplementary Fig. 7) and that the electrode grids (30 μ m lines with 40 μ m spacing) were successfully transferred. When the matrix was turned on the individual pixels could be clearly distinguished (Fig. 3f). Upon 30% strain the broadening/narrowing of the AgNW tracks is clearly visible together with the light up pixels (Fig. 3g). When the matrix is viewed in the dark and stretched one can see how the distance and shape of the pixels are evolving with strain (Fig. 3h). It should be noted that the brightness of the pixels decrease with the strain, especially above 30% strain. However, most of the brightness is regained upon relaxation as the resistance of the tracks goes down again.

## Conclusions

In summary, we have presented a generic method for fabricating high resolution intrinsically stretchable electronics from high aspect ratio nanomaterials. The resulting conductors are the best reported so far with respect to dimensions and performance (Fig. 1a), and large scale micropatterns can be achieved without major defects. Another strong point of the method is the low material consumption, which allows for the use of even more expensive nanomaterials than AgNWs. Two inch substrates were used in this work, however, there are no fundamental limitations for scaling up the approach to at least 4 inch substrates and it might also be possible to reuse the patterned membranes. Further, the patterning is free of organic solvents which allowed us to construct multilayer devices without material compatibility concerns, an advantage not to be underestimated when dealing with elastomers. Based on the strengths of the method, a three layer high resolution EL matrix display was developed. The 70 μ m pixel size of our display compares well with previously reported displays, where the best one has 180 μ m pixel size^4^, while the others are in the mm range^30^,^32^,^33^,^34^. Although the presented display is small, there are no fundamental limitations preventing the upscaling and addressing of it with the proper driving circuitry. In conclusion, we anticipate that the method can be used to construct a wide array of new miniaturized stretchable electronic devices including displays, electronic skin and neuroprosthetics.

## Methods

### Membrane patterning

Hydrophilic PVDF membranes (0.22 μ m pore size, Millipore) were baked on a hotplate (100 °C, 10 min). Photoresist ma-N 490 (micro resist technology GmbH) was spin coated onto the membranes (6000 rpm, 60 s). The coated membranes were dried in vacuum (200 mbar, 1 h), followed by softbake in an oven (80 °C, 1 h). The membranes were UV exposed (9 ×  20 s, 10 s rest) in a mask aligner (Suss MA 4, 10 mW at 360 nm), developed (2.5–3.5 min, ma-D 332 S, micro resist technology GmbH) and gently rinsed in DI water (1 min). The membranes were dried and hardbaked (80 °C, 10 min).

### Nanowire filtration

The patterned membranes were exposed to O2-plasma (100 W, 4 min) and soaked in DI water containing detergent (Triton X100, Sigma Aldrich). AgNWs (60 nm diameter, 10 μ m long, Sigma Aldrich) were dispersed in ethanol, vortexed and briefly ultrasonicated. Immediately prior to filtration, the AgNWs were diluted in 15 ml DI water and vortexed. The dispersion was filtered through the membrane and the membrane with the deposited AgNWs was dried on a hotplate (50 °C, 10 min). The surface coverage of AgNWs on the patterned membrane area was approximately 0.17 mg/cm^2^.

### Nanowire transfer

Silicon wafers were O2-plasma activated (300 W, 2 min) and vapor phase silanized in vacuum (Trichloro(1H,1H,2H,2H-perfluorooctyl)silane, 1 h, Sigma Aldrich). The silanized wafers were spin coated with a release agent (Ease Release 205, 3000 rpm, 20 s, Mann Release Technologies). PDMS (Sylgard 184, Dow Corning) was spin coated (500 rpm, 30 s) and semi-cured on a hotplate (65 °C, 10 min). Membranes with deposited AgNWs were baked (100 °C, 10 min) and put in contact with the PDMS layer under pressure and heat (100 °C, 5 min). The membranes were peeled of leaving the AgNWs on the PDMS surface. SEM images were taken with a Zeiss SUPRA 50 VP (acceleration voltage 5–10 kV).

### Electromechanical characterization

Samples of 20 mm length were clamped in a tensile testing machine (DO-FB0.5TS, Zwick/Roell). Electrical contact was made with copper pads covered with eutectic gallium–indium (Sigma Aldrich). The resistance was measures at 4 Hz with a digital multimeter (Agilent 34401A). Maximum strain tests were performed at 0.5 mm/s while cycling tests were performed at 2 mm/s.

### EL matrix fabrication

PDMS was spin coated (1000 rpm, 30 s) onto a silanized substrate and semi-cured on a hotplate (65 °C, 10 min). The first AgNW layer (40 μ g/cm^2^) was transferred as described above. The contacts were masked off with 25 μ m thick Teonex Q51 foil. Electroluminescent phosphor particles (< 7 μ m, D447S, Shanghai Keyan Phosphor Technology Co., Ltd) was put through a 20 μ m sieve and mixed in a 1:1 weight ratio with PDMS using a THINKY ARE-250 Mixer. The mixture was spin coated (5000 rpm, 60 s), removed from the contacts and semi-cured on a hotplate (65 °C, 10 min). The second layer of AgNWs were transferred and the contacts were masked off again. Finally, a PDMS layer was spin coated (1000 rpm, 30 s), removed from the contacts and cured (100 C, 1 h). Copper foil contacts were glued on with silver epoxy (8330S, MG Chemicals, 100 C, 1 h cure).

## Additional Information

**How to cite this article**: Tybrandt, K. *et al.* Multilayer Patterning of High Resolution Intrinsically Stretchable Electronics. *Sci. Rep.* **6**, 25641; doi: 10.1038/srep25641 (2016).

## Supplementary Material

Supplementary Information
