# Supplementary material for: Multilayer Patterning of High Resolution Intrinsically Stretchable Electronics
Source: Sci Rep. 2016 May 9;6:25641. doi: 10.1038/srep25641 (PMC4860633; doi:10.1038/srep25641)
Supplement: Supplementary Information [file srep25641-s1.pdf]

Supplementary Information

Multilayer Patterning of High Resolution Intrinsically Stretchable Electronics

Klas Tybrandt, Flurin Stauffer, Janos Vörös*

Institute for Biomedical Engineering, ETH Zurich

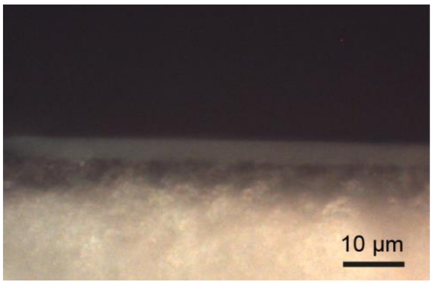

Supplementary Figure 1. Side view of a cut photoresist covered PVDF membrane. The maN-490 photoresist layer is visible on top of the membrane and has a thickness of ~4 µm.

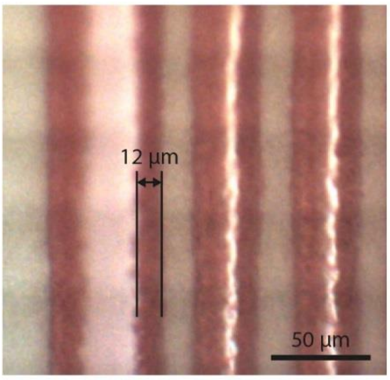

Supplementary Figure 2. Micrograph of ma-P 1275 HV photoresist patterned on top of a PVDF membrane. The sidewalls of the developed resist are far from vertical and narrow membrane areas are noticeably darker from resist residues.

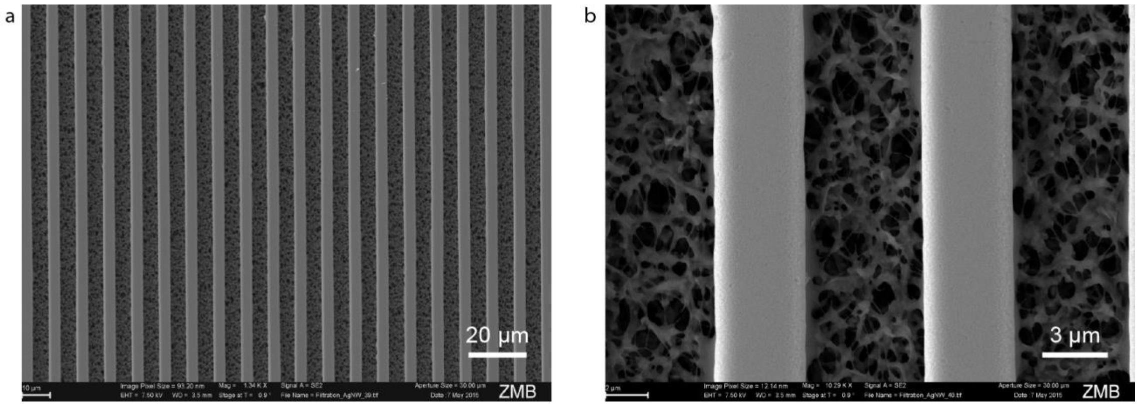

Supplementary Figure 3. SEM images of resist lines on top of a PVDF membrane. **a)** The lines are regular with no major defects. **b)** The actual width of the resist lines is ~4 µm. The open pores of the PVDF membrane are clearly visible in between the lines.

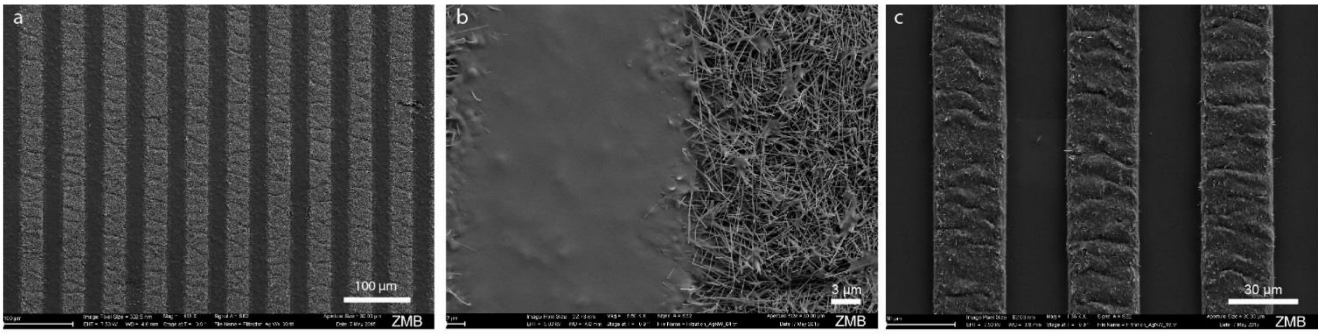

Supplementary Figure 4. SEM images of AgNWs transferred onto a semicured PDMS substrate. **a)** The transferred AgNW pattern show no defects over the inspected area. **b)** At the edge of the AgNW lines the nanowires are embedded in the PDMS substrate. Within the lines there are areas where the PDMS has penetrated the AgNW layer. This partial penetration likely improves the transfer of the AgNWs from the membrane to the substrate by increasing the adhesion of the AgNWs to the PDMS surface. **c)** When a less cured PDMS substrate is used the PDMS can fully penetrate the AgNW film, which makes it hard to electrically contact the AgNWs.

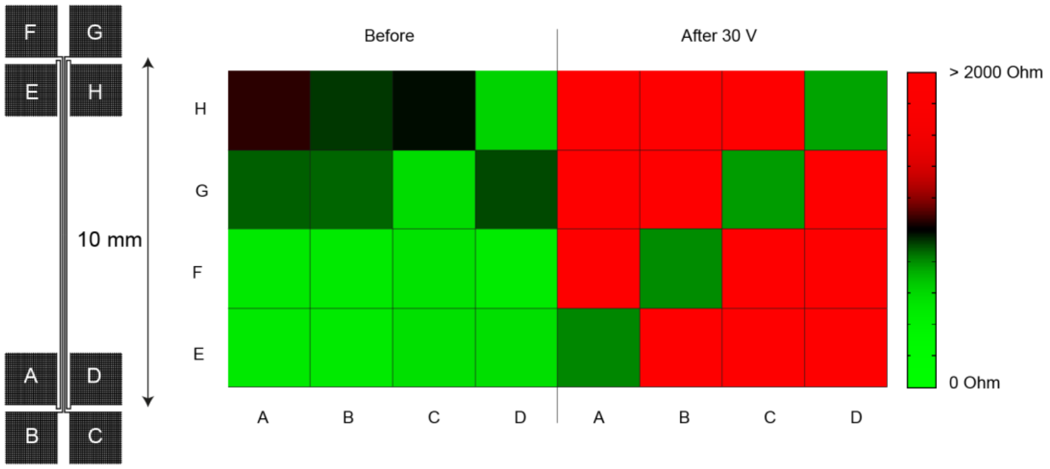

Supplementary Figure 5. Treatment of shorted 20 µm lines. The test structure to the left comprises four 20 µm wide AgNW lines with 20 µm separation. Initially, the lines are shorted because of AgNW residues in between the lines. By sequentially applying 30 V between the shorted lines, the shorts were melted away and the lines became electrically insulated from each other. The change in resistance between the test pads after this treatment is shown to the right.

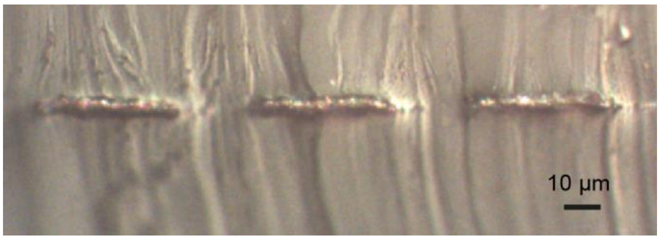

Supplementary Figure 6. Side view of AgNW tracks embedded in PDMS. The AgNW tracks visible in the cut were approximately 3 µm thick. This is in line with previous SEM and AFM measurements of similar tracks.

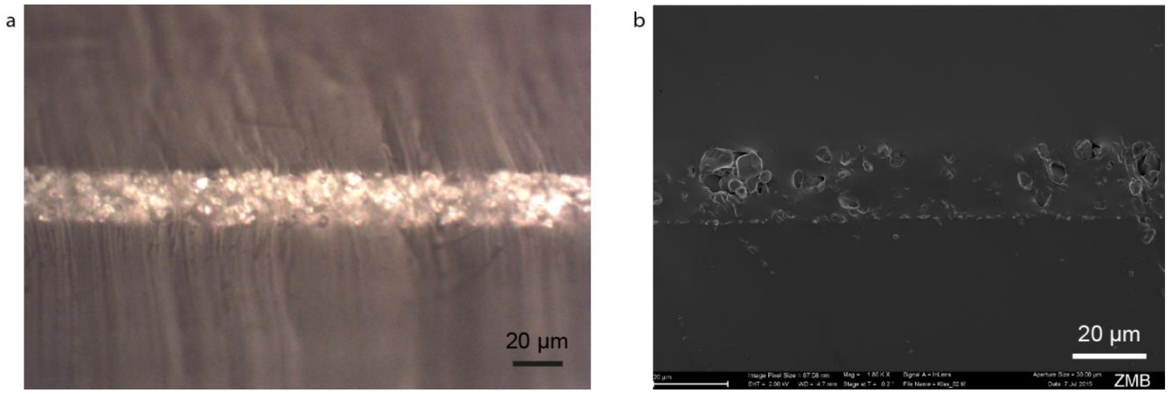

Supplementary Figure 7. Side view of EL particles embedded in PDMS. (a) The micrograph shows that the thickness of the EL particle layer is approximately 20 µm. (b) Individual EL particles can be distinguished in the SEM image of the cut. Traces of an AgNW layer are also visible beneath the EL particle layer.
